# Local X-ray magnetic circular dichroism study of Fe/Cu(111) using a tunneling smart tip

**DOI:** 10.1107/S1600577515023383

**Published:** 2016-01-28

**Authors:** Andrew DiLullo, Nozomi Shirato, Marvin Cummings, Heath Kersell, Hao Chang, Daniel Rosenmann, Dean Miller, John W. Freeland, Saw-Wai Hla, Volker Rose

**Affiliations:** a Center for Nanoscale Materials, Nanoscience and Technology Division, Argonne National Laboratory, 9700 South Cass Avenue, Argonne, IL 60439, USA; b Advanced Photon Source, Argonne National Laboratory, 9700 South Cass Avenue, Argonne, IL 60439, USA; c Nanoscale and Quantum Phenomena Institute, Physics and Astronomy Department, Ohio University, Athens, OH 45701, USA

**Keywords:** synchrotron X-ray scanning tunneling microscopy, smart tip, XMCD, chemical contrast

## Abstract

Localized spectroscopy with simultaneous topographic, elemental and magnetic information is presented. A synchrotron X-ray scanning tunneling microscope has been employed for the local study of the X-ray magnetic circular dichroism at the Fe *L*_2,3_-edges of a thin iron film grown on Cu(111). Polarization-dependent X-ray absorption spectra have been obtained through a tunneling smart tip that serves as a photoelectron detector. In contrast to conventional spin-polarized scanning tunneling microscopy, X-ray excitations provide magnetic contrast even with a non-magnetic tip. Intensity variations in the photoexcited tip current point to chemical variations within a single magnetic Fe domain.

## Abstract 2

A tunneling smart tip of a synchrotron X-ray scanning tunneling microscope provides simultaneously localized topographic, elemental and magnetic information.

## 1. Introduction   

The real-space observation of magnetic structure using scanning probe microscopy (SPM) methods (Wiesendanger, 2009 ▸; Oka *et al.*, 2014 ▸, Heinrich *et al.*, 2015 ▸) or synchrotron-based microscopy (Nolting, 2010 ▸; Cheng & Keavney, 2012 ▸; Fischer, 2015 ▸) continues to have a tremendous impact on our understanding of nanomagnetism. Spin-polarized scanning tunneling microscopy (SP-STM) is sensitive to the spin orientation of tunneling electrons, while magnetic force microscopy (MFM) detects the forces between a magnetic sample surface and a magnetic tip. Although these methods provide high spatial resolution, they lack direct chemical contrast. Photoemission electron microscopy (PEEM), on the other hand, can provide chemical as well as magnetic sensitivity. However, the spatial resolution is limited by the spread of photoelectron emission angles.

In order to overcome these limitations, groups around the world have been developing instruments that combine synchrotron radiation with the high spatial resolution of different SPM variants (Okuda *et al.*, 2005 ▸; Saito *et al.*, 2006 ▸; Scheler *et al.*, 2009 ▸; Rose & Freeland, 2010*a* ▸; Fauquet *et al.*, 2011 ▸; Pilet *et al.*, 2012 ▸; Chan *et al.*, 2013 ▸; Suzuki, 2015 ▸; Slobodskyy *et al.*, 2015 ▸). Recently, synchrotron X-ray scanning tunneling microscopy (SX-STM) showed the capability to obtain elemental contrast with a lateral spatial resolution of only 2 nm and sensitivity at the limit of single-atomic height (Shirato *et al.*, 2014 ▸). The SX-STM technique utilizes the energy-dependent absorption of X-rays to obtain information about the elemental composition of a sample. While a specialized probe tip is tunneling above a sample surface, core electrons are excited in the absorption process into empty states above the Fermi energy. At the same time, secondary electrons are ejected from the sample surface (Rose *et al.*, 2008 ▸). Both contributions, *i.e.* X-ray excited tunneling and photoejected electrons, modulate the conventional tunnel current and consequently provide chemical contrast (Okuda *et al.*, 2009 ▸; Rose *et al.*, 2013 ▸). However, in order to extract the real chemical information it is essential to separate the topographic signal from the X-ray excited currents through filtering (Wang *et al.*, 2013 ▸). Additionally, specialized smart tips are used to enhance the spatial resolution of the method by restricting the electron detection to the very tip apex (Akiyama *et al.*, 2007 ▸; Saito *et al.*, 2007 ▸; Rose *et al.*, 2011 ▸; Shirato *et al.*, 2014 ▸; Yan *et al.*, 2015 ▸).

The use of polarized synchrotron X-rays provides the opportunity to probe the magnetic properties of a sample surface in addition to gathering the aforementioned chemical information (Stöhr *et al.*, 1998 ▸). Hence, the use of polarized X-rays in SX-STM has been proposed before and feasibility experiments were carried out (Rose & Freeland, 2010*b* ▸; Rose *et al.*, 2012 ▸). However, so far the tip could only be located in the far field, several hundred nanometers outside the quantum mechanical tunneling regime, which makes it impossible to obtain high-resolution information. In this paper, we present the measurement of the local X-ray magnetic circular dichroism (XMCD) signal of a magnetic domain in an iron film using a non-magnetic tip that is actually tunneling over the sample surface. This means that the separation between the tip, *i.e.* detector, and the sample is typically less than 1 nm. The goal is to combine the spin sensitivity of X-ray magnetic circular dichroism with the locality of scanning tunneling microscopy. This capability has only recently become available through the development of a topographic filter (Wang *et al.*, 2013 ▸) that removes X-ray excited current contributions from the STM feedback signal and therewith provides stable tunneling conditions even under X-ray illumination.

## 2. Principle of localized XMCD by a smart tip   

**Figure 1:**
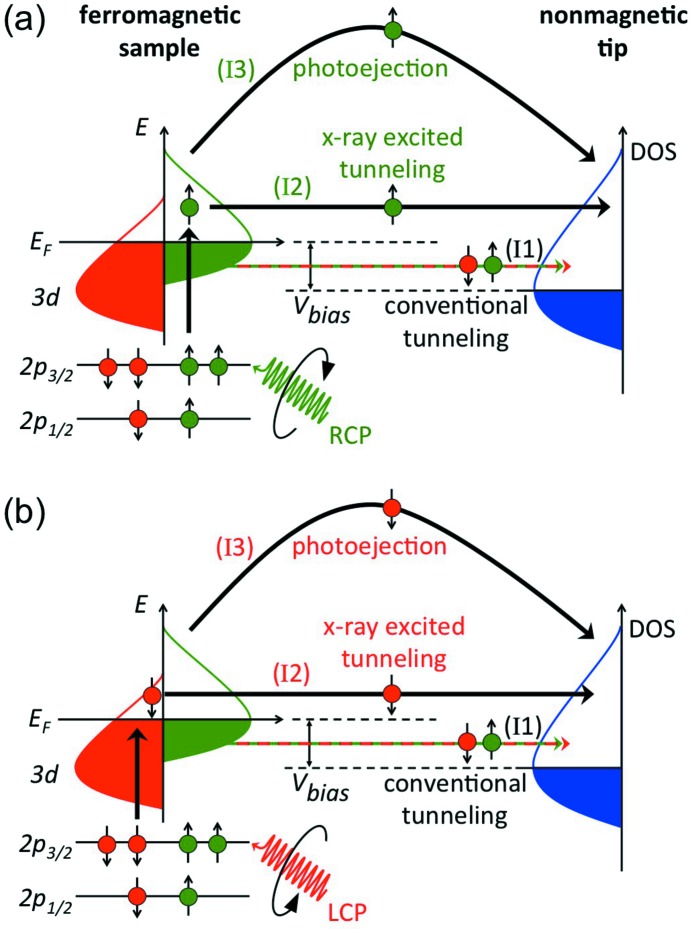
(*a*) Schematic representation of X-ray enhanced magnetic contrast in SX-STM. In the ferromagnetic sample, right circularly polarized (RCP) X-rays predominately excite spin-up electrons, which can modulate the conventional tunnel current (I1) through spin-polarized X-ray excited tunneling (I2) and photoejected electrons (I3) that reach the nonmagnetic tip. The density of states (DOS) of tip and sample are shifted by the tunneling bias *V*_bias_ of the SX-STM. (*b*) Changing to left circularly polarized (LCP) X-rays allows exciting electrons of opposite spin, while the conventional tunnel current (I1) remains unchanged.

Fig. 1 ▸ depicts a model of the origin of the magnetic contrast in SX-STM for a tunneling tip under X-ray illumination of a ferromagnetic sample. Magnetic properties of 3*d* transition metals are determined primarily by their *d* valence electrons. The core level is split into *p*_3/2_ and *p*_1/2_ states, where spin and orbit are coupled parallel and antiparallel, respectively. Right circularly polarized (RCP) and left circularly polarized (LCP) photons transfer an opposite angular momentum to the excited photoelectrons, and mostly electrons with opposite spins are created in the two cases. Since spin flips are forbidden in electric dipole transitions, spin-up photoelectrons from the *p* core shell can only be excited into spin-up *d* hole states (Fig. 1*a* ▸). Subsequently, these electrons can tunnel into the nonmagnetic tip, modulating the conventional tunnel current. At the same time, spin-up electrons with sufficiently high energy can overcome the work function of the sample and are ejected. Some of these ejected electrons can be detected at the tip. Likewise, LCP X-rays predominantly generate spin-down electrons (Fig. 1*b* ▸) causing similar characteristic currents. Theoretical sum rules relate the integrated difference signal in photoabsorption between LCP and RCP X-rays at the 2*p* absorption edges to the ground-state magnetic moment of the 3*d* transition metals (Thole *et al.*, 1992 ▸; Stöhr, 1999 ▸). In the case of SX-STM, in contrast to conventional spin-polarized STM measurements (Smith, 2006 ▸), a magnetic tip is not required, because the modulation of the conventional tunnel current is directly controlled by the polarization of the X-rays that interact with the sample. Therefore, the tip does not have to act as a spin filter.

**Figure 2:**
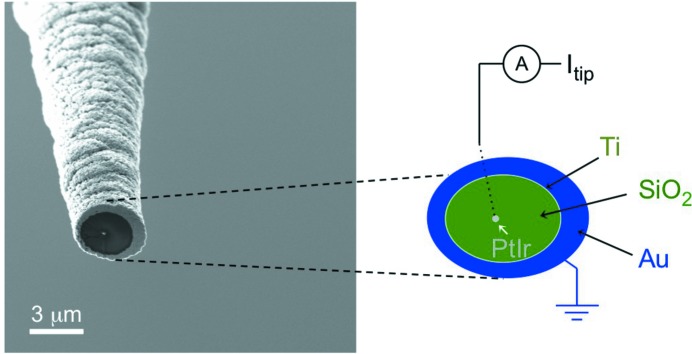
Scanning electron microscopy image of a nonmagnetic smart tip, which serves as detector for photoexcited electrons from the sample. The inner PtIr core constitutes the tunneling tip, which provides the tip current *I*_tip_. It is coated with insulating and metallic films except at the tip apex. The outer metallic layer is grounded to avoid potential charging through ejected electrons that travel from the sample to the sidewalls of the tip.

While electron tunneling is generally highly localized, ejected electrons can be generated by the entire sample area that is illuminated by the X-ray beam. Consequently, photoejected electrons can deteriorate the spatial resolution in SX-STM. Hence, in order to obtain localized information, the background of photoejected electrons has to be minimized by using a specialized smart tip that focuses the electron detection to only the tip apex. Fig. 2 ▸ shows a scanning electron micrograph of a nanofabricated PtIr tip, coaxially coated with layers of SiO_2_, Ti and Au, as used in our experiment. The smart tip was fabricated by electrochemically etching a Pt_90_Ir_10_ wire of 250 µm outer diameter in 1.5 *M* calcium chloride solution. After cleaning with ionized water, acetone and alcohol, and subsequent drying with nitrogen gas, the tip was introduced into a sputtering system with three DC magnetron guns used to deposit the coaxial trilayer. During deposition the tip was rotated at 20 r.p.m. to aid in film uniformity. The trilayer consists of 1.5 µm SiO_2_ insulating film, an intermediate 20 nm-thin seed layer of Ti and a final Au film, 0.5 µm thick. At the final stage of the smart tip fabrication the tip apex was exposed by removing the coated films using focused ion beam (FIB) milling. While the oxide layer of the smart tip minimizes the background caused by photoejected electrons, the outer metallic Ti-Au layers are grounded to avoid undesired charging effects that could disturb the tunneling of the tip over the sample surface.

## 3. Topography, chemistry and XMCD of Fe/Cu(111)   

**Figure 3:**
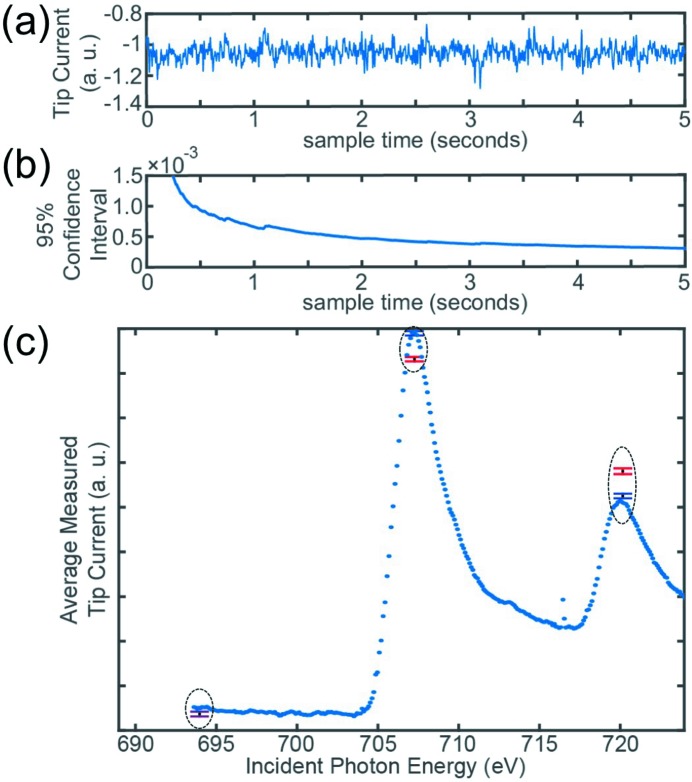
(*a*) Representative time series of the measured tip current for one image pixel at fixed polarization and photon energy. (*b*) 95% confidence interval for this pixel as a function of sampling time. (*c*) Examples of the average tip current including standard deviations for three different photon energies (dotted circles); LCP: blue; RCP: red. The LCP reference spectrum recorded with a stationary but tunneling tip exhibits Fe *L*_2,3_ absorption peaks.

For this experiment we used the second-generation synchrotron X-ray scanning tunneling microscope (Cummings *et al.*, 2012 ▸), SX-STM V2, at the soft X-ray beamline 4-ID-C of the Advanced Photon Source at Argonne National Laboratory (Freeland *et al.*, 2002 ▸). A 15 nm-thick iron film was grown *in situ* on a clean Cu(111) single crystal. Sample growth as well as the subsequent measurements were carried out at room temperature. The smart tip was mapped over the sample surface using a feedback system that can separate topographic from X-ray induced current contributions (Wang *et al.*, 2013 ▸). At each image pixel, the tip current *I*_tip_ was recorded in real time for 5 s (Fig. 3*a* ▸) for selected photon energies at both X-ray polarizations, LCP and RCP. This approach provides data with high statistical confidence (Fig. 3*b* ▸) and therewith reduces potential artifacts. However, previously imaging with elemental contrast and dwell times of only tens of microseconds has been demonstrated (Shirato *et al.*, 2014 ▸). The statistical 95% confidence interval of the mean tip current drastically improves over the first 2 s of sampling and then asymptotically advances further up to 5 s sampling time. In Fig. 3(*c*) ▸ we show examples of the average tip current *I*_tip_ over a selected image pixel for three different photon energies, the pre-edge at 695 eV as well as the Fe *L*_3_- and *L*_2_-edges. The tip current was recorded for both LCP (blue) and RCP (red) radiation. The error bars represent the standard deviations of the particular measurement. The inversion of the intensities at the *L*_3_- and *L*_2_-edges is indicative of the ferromagnetism of the Fe sample. A complete reference absorption spectrum of the sample is also shown in Fig. 3(*c*) ▸ as a guide. It exhibits the characteristic Fe *L*_3_ and Fe *L*_2_ absorption peaks of the metallic iron film at 707 and 720 eV, respectively.

**Figure 4:**
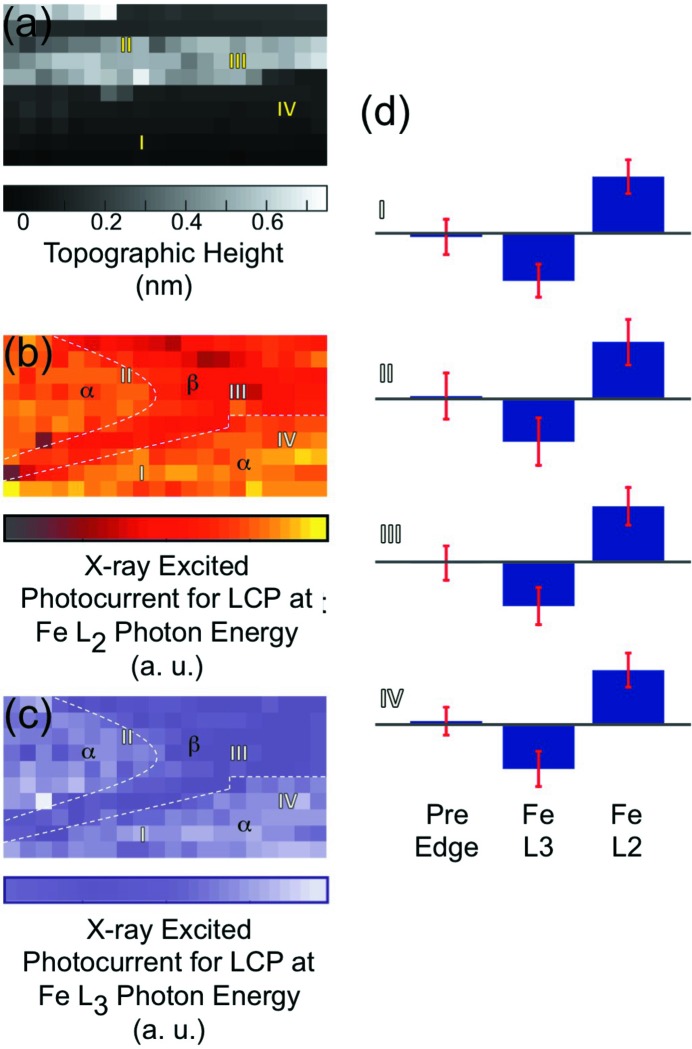
(*a*) Topographic image measured concurrently with the magnetic contrast (200 nm × 100 nm). (*b*) Map of the X-ray excited current obtained with LCP X-rays at the Fe *L*_2_-edge. The dotted lines separate two different domain-type areas α and β. (*c*) X-ray excited currents for LCP X-rays at the Fe *L*_3_-edge. (*d*) XMCD obtained from the difference of LCP and RCP spectra shown for a set of the four points I–IV marked in the maps.

Obtaining spectroscopic information at different surface positions allows generating maps of the surface topography, chemistry and magnetic properties. Fig. 4(*a*) ▸ shows a 200 nm × 100 nm topographic map of the sample surface. The topographic map exhibits a flat terrace (see pixels I, IV) and a step feature with an average height of about 0.5 nm (see II, III). In Fig. 4(*b*) ▸ we present the simultaneously obtained map of the X-ray excited photocurrent for the Fe *L*_2_-edge with LCP X-rays. The localization of the SX-STM measurement becomes clearly evident in the observed image contrast. The map yields two different domain-type areas, α and β, with a photocurrent intensity ratio *I*(α/β) = 1.5. Interestingly, the shape of the α and β areas is clearly different from that observed in the surface topography, supporting that the chemical contrast is not governed simply by the height of a material. Instead, the first atomic surface layer determines the chemical contrast (Shirato *et al.*, 2014 ▸). Likewise, the same contrast can be observed at the Fe *L*_3_ absorption edge (Fig. 4*c* ▸). The reduced intensity in the β surface region is most likely caused by the onset of oxidation, which shifts the absorption peak to higher energies, but further studies are required. Fig. 4(*d*) ▸ shows the local dichroism for selected pixels I–IV. The difference LCP–RCP provides a clear proof of XMCD at the Fe *L*_2,3_-edges (707 and 720 eV). As expected, a dichroism signal is absent at the pre-edge (695 eV). Overall, the analysis of the XMCD signal for the whole scanned area yields uniform intensities, regardless of the topographic step features (*cf.* Fig. 4*a* ▸) or the chemical contrast (*cf.* Figs. 4*b* and 4*c* ▸). The mean dichroism intensity ratio for the areas α and β amounts to 1.0. This indicates the presence of a single magnetic domain in the scan area. The chemical variation in the surface area β does not impact the magnetic properties, which implies that the degree of oxidation is small and that a full ferrimagnetic iron oxide phase has not yet formed.

## 4. Conclusion   

We have shown the capability to obtain magnetic information from a surface through a specialized, but non-magnetic, tunneling tip of an STM. Illumination of the sample with polarized monochromatic X-rays yields excited electrons that modulate the conventional tunneling current. The distinct advantage of the combination of X-rays and STM lies not only in the ability to localize spins but also in its capability to determine topography and chemistry simultaneously as well as the potential to measure the actual size of magnetic moments using the dichroism effect. It is anticipated that this capability will open up the path to unique insight into nanoscale magnetism at surfaces, complementary to conventional PEEM measurements.
